# Serum lipidomics reveals phosphatidylethanolamine and phosphatidylcholine disorders in patients with myocardial infarction and post-myocardial infarction-heart failure

**DOI:** 10.1186/s12944-023-01832-0

**Published:** 2023-05-20

**Authors:** Jidong Rong, Tianmu He, Jianyong Zhang, Zhixun Bai, Bei Shi

**Affiliations:** 1 Department of Cardiology, Affiliated Hospital of Zunyi Medical University, Zunyi, China; 2 School of Basic Medical Sciences, Guizhou medical University, Guiyang, China; 3 College of pharmacy, Zunyi medical University, Zunyi, China; 4 Department of Internal Medicine, The Second Affiliated Hospital of Zunyi Medical University, Zunyi, China

**Keywords:** Myocardial infarction, Heart failure, Lipidomics

## Abstract

**Background:**

Myocardial infarction (MI) and post-MI-heart failure (pMIHF) are a major cause of death worldwide, however, the underlying mechanisms of pMIHF from MI are not well understood. This study sought to characterize early lipid biomarkers for the development of pMIHF disease.

**Methods:**

Serum samples from 18 MI and 24 pMIHF patients were collected from the Affiliated Hospital of Zunyi Medical University and analyzed using lipidomics with Ultra High Performance Liquid Chromatography and Q-Exactive High Resolution Mass Spectrometer. The serum samples were tested by the official partial least squares discriminant analysis (OPLS-DA) to find the differential expression of metabolites between the two groups. Furthermore, the metabolic biomarkers of pMIHF were screened using the subject operating characteristic (ROC) curve and correlation analysis.

**Results:**

The average age of the 18 MI and 24 pMIHF participants was 57.83 ± 9.28 and 64.38 ± 10.89 years, respectively. The B-type natriuretic peptide (BNP) level was 328.5 ± 299.842 and 3535.96 ± 3025 pg/mL, total cholesterol(TC) was 5.59 ± 1.51 and 4.69 ± 1.13 mmol/L, and blood urea nitrogen (BUN) was 5.24 ± 2.15 and 7.20 ± 3.49 mmol/L, respectively. In addition, 88 lipids, including 76 (86.36%) down-regulated lipids, were identified between the patients with MI and pMIHF. ROC analysis showed that phosphatidylethanolamine (PE) (12:1e_22:0) (area under the curve [AUC] = 0.9306) and phosphatidylcholine (PC) (22:4_14:1) (AUC = 0.8380) could be potential biomarkers for the development of pMIHF. Correlation analysis showed that PE (12:1e_22:0) was inversely correlated with BNP and BUN, but positively correlated with TC. In contrast, PC (22:4_14:1) was positively associated with both BNP and BUN, and was negatively associated with TC.

**Conclusions:**

Several lipid biomarkers were identified that could potentially be used to predict and diagnose patients with pMIHF. PE (12:1e_22:0) and PC (22:4_14:1) could sufficiently differentiate between patients with MI and pMIHF.

## Background

Heart failure (HF) is a complex clinical syndrome characterized by abnormal cardiac structure and function, which can impede ventricular filling and the discharge of blood into systemic circulation. Fibrosis is one of the most important contributors to tissue stiffness and dysfunction as it leads to low ventricular filling or reduced pumping of blood [1, 2]. The prevalence of HF has increased by 44% in the past 15 years and it is estimated that 1.3% (13.7 million) of adults in China are affected by it [3]. Myocardial infarction (MI), one of the early manifestations of HF, causes myocardial cell necrosis, which exacerbate HF. The incidence of post-MI-heart failure (pMIHF) among patients hospitalized for MI ranges from 14 to 36% according to various studies [4]. Moreover, comorbidities including coronary artery disease (CAD), diabetes, and hypertension may be crucial in the development and management of pMIHF. Despite advances in medical therapy, patients with HF generally have a poor prognosis and high mortality rates [5].

Evaluation of myocardial infarction, atrial or ventricular arrhythmias, and deteriorating renal function should be the focus of the initial heart failure examination. The workups should at a minimum include an electrocardiogram, assessment of type b-natriuretic Peptide (BNP) levels, chest x-rays, troponin level assessment, and a transthoracic echocardiogram [6]. HF is often associated with a decrease in the fraction of left ventricular volume ejected per beat (LVEF) [7], and thus, early detection is crucial for HF treatment. BNP levels have been found to be proportional to the severity of HF and are an established HF predictor [8]. Therefore, screening similar early biomarkers could be helpful for the clinical diagnosis and prognosis of pMIHF patients [9]. However, the clinical application of biomarkers is limited by detection methods, unknown responses to drugs, and susceptibility to noncardiogenic factors [10]. Current biomarkers are mainly focused on diagnosis and evaluation of the prognosis of HF, and biomarkers capable of provide early warning are limited. Thus, it is crucial to identify novel biomarkers that could provide an early warning of pMIHF development.

Cardiac lipid overload leads to increased destructive levels of reactive oxygen species (ROS). These disrupt normal mitochondrial structures by changing the activity of several important proteins that help control mitochondrial size and shape, which may impair energy production and compromise cardiac function.

Plasma lipidomic signatures reflect altered characteristics of cardiac lipid metabolism, which can lead to a predisposition to HF in vivo. Moreover, metabolic disorders of lipids are related to HF accompanying CAD and diabetes [11]. Lipid biomarkers, with great potential for pMIHF diagnosis have been identified. Metabolomics is a novel tool that can be used to systematically explore the metabolic program of organisms. Due to its high-throughput nature, it is ideal for screening HF-related biomarkers [12]. Lipidomics, an important branch of metabolomics, is a powerful tool to detect early lipid markers. Lipidomics can facilitate biomarker screening, thereby helping to elucidate the pathophysiology of HF [13, 14]. The biomarkers ceramide (Cer) (16:0), phosphatidylcholine (PC) (32_0), oxidized cholesterol (7-ketocholesterol), lysophosphatidylcholine (LPC) (18:2), and cholesteryl ester (18:1) were found significantly associated with BNP, left ventricular ejection fractions, and HF risk [11, 15, 16]. However, early detection lipid biomarkers for pMIHF diagnosis are still unexplored. Therefore, lipidomics could be utilized to elucidate HF pathogenesis and identify new biomarkers for HF complicated by MI.

In this study, a ultra-high performance liquid chromatography (UHPLC)-Q-Exactive MS lipidomic approach was adopted to screen for early warning biomarkers in patients with pMIHF. First, the endogenous serum differential metabolites of patients with pMIHF were screened, and a metabolic network map of the HF development process was constructed. Univariate and multiple factor statistical evaluation methods were used to evaluate lipid biomarkers in patients with pMIHF.

## Materials and methods

### Patient population

This study was approved by the Ethical Evaluation Committee of the Affiliated Hospital of Zunyi Medical University (ZMU [2022] 1-177) and was designed using a case-control method. The patients provided informed consent for lipid analysis. The study included 42 patients who were diagnosed with either MI (18) or pMIHF (24). MI was defined according to a previous description, and pMIHF was defined as newly diagnosed HF after admission [17]. Plasma was collected on the first day after admission. Informed consent was obtained from all subjects prior to receiving percutaneous coronary intervention (PCI) and blood sample collection.

### Lipidomics analysis of serum samples

Lipids in the serum samples were detected by Shanghai Applied Protein Technology Inc.

#### Total lipid extraction

Human serum samples were vortexed after adding 200 µL of water and 800 µL of MTBE. Subsequently, 240 µL of precooled methanol was added to the mixture and vortexed for 30 s. The mixture was subjected to ultrasound for 20 min at 4 ℃ and left undisturbed for 30 min at room temperature. The solution was then centrifuged for 15 min at 14,000 g (10 °C), and the upper organic solvent layer was collected and dried under nitrogen. Subsequently, 200 µL of 90% isopropanol/acetonitrile solution was added for re-solubilization, followed by vortexing. Thereafter, 90 µL of the re-solution solution was collected and centrifuged for 15 min at 14,000 g (10 °C), and the supernatant was collected for LC-MS analysis.

#### Metabolomics analysis

The samples were analyzed using an LC-MS/MS system (UHPLC, Nexera LC-30 A; MS, Q Exactive, Thermo Scientific™). The analytical conditions were as follows: UHPLC: column, Waters ACQUITY UPLC CSH C18 (1.7 μm, 2.1 mm × 100 mm); column temperature, 45 °C; flow rate, 300 µL/min; solvent system, (acetonitrile/water = 6:4, V1/V2, A): (acetonitrile/isopropanol = 1:9, V1/V3, B); and gradient program: 0–2 min, 30% B, followed by 2–25 min, 30% B increasing linearly to 100%, and finally 25–35 min, 30% B. The autosampler temperature was 10 ℃.

UHPLC was used to separate the samples, and MS/MS was used to analyze the results. Electrospray ionization(ESI) source conditions: heater temperature, 300 °C; sheath gas flow rate, 45 arb; aux gas flow rate, 15 arb; sweep gas flow rate, 1 arb; spray voltage, 3.0 kV; capillary temperature, 350 °C; S-lens RF level, 50%; and MS1 scan ranges, 200–1800. The mass-to-charge ratios of the lipid molecules and lipid fragments were determined according to the following conditions: ten fragment profiles were acquired after each full scan (MS2 scan, HCD). MS1 and MS2 had a resolution of 70,000 at M/Z 200 and 17,500 at M/Z 200, respectively.

#### Raw data preprocessing

Raw data were processed using the LipidSearch software (Thermo Scientific™) for peak identification, peak extraction, and lipid identification (secondary identification) of lipid molecules and internal standards. The main parameters were: precursor tolerance, 5 ppm; product tolerance, 5 ppm; and product ion threshold, 5%. Data were then extracted for quality evaluation and analysis. Quantitative statistics, composition analysis, and differential analysis were performed for lipid data analysis.

#### Multivariate statistical analysis

The SIMCA 14.1 software (Umea, Sweden) was used to analyze the final datasets, which contains information about the peak number, sample name and normalized peak area of multivariate analysis. Logarithmically transforming and scaling the data minimized the effect of noise and high variance. Principal component analysis (PCA) was performed to visualize the sample distribution. Partial least-squares discriminant analysis (PLS−DA) was used to observe the distribution of groups.

#### Potential biomarker screening

Supervised orthogonal projections to latent structures-discriminate analysis (OPLS−DA) was performed to visualize group separation and identify significantly differential metabolites. To check the robustness and predictive ability of the OPLS−DA model, 200 permutations were performed. Potential biomarkers screening: variable importance in the projection (VIP) > 1, *P* < 0.05. Volcano plots, hierarchical clustering, and heat maps were used to directly observe the profile of potential biomarkers and evaluate the rationality of the differential lipids. MetaboAnalyst [18] (http://www.metaboanalyst.ca/) was used for metabolic pathway enrichment analysis associated with potential biomarkers.

### Integration analysis of biomarkers

To explore the potential biomarkers, analyses of univariate receiver operating characteristic (ROC) and correlation of key biomarkers post-HF-MI were performed using GraphPad Prism 9.0. The top five potential biomarkers with highest fold change values in up- and down-regulation groups were identified. Potential biomarkers involved in key metabolic processes were also screened. The Pearson correlation coefficient was used to analyze the correlation between two variables. Furthermore, correlation analysis was performed to elucidate the five potential biomarkers with higher AUC for TC, BNP, and BUN levels.

### Statistical analyses

The baseline patient characteristics were summarized as mean ± standard deviation (SD) for continuous variables and frequency for categorical variables. All statistical analyses were performed with GraphPad Prism software (version 9.0). Data are presented as the mean ± SD or ± SEM. Student’s *t*-test and one way analysis of variance (ANOVA) were used for comparisons. A *p* value of less than 0.05 was considered statistically significant.

## Results

### Baseline characteristics

Initially, 42 subjects, 18 with MI and 24 with pMIHF, were enrolled in the study. The baseline characteristics and laboratory data are presented in Table 1. Notable differences were observed between the characteristics of the patients with MI and pMIHF. The mean age of patients with pMIHF (64.38 ± 10.89) was higher (*P* < 0.05) than that of patients with MI (57.83 ± 9.28). The BNP (3535.96 ± 3025.35 pg/mL) and BUN (7.20 ± 3.49 mmol/L) levels were increased in patients with pMIHF compared to those in patients with MI (BNP = 328.5 ± 299.842 pg/mL, BUN = 5.24 ± 2.15 mmol/L) (*P* < 0.05). However, the TC levels in patients with pMIHF (4.69 ± 1.13 mmol/L) were lower than that in patients with MI (5.59 ± 1.51 mmol/L) (*P* < 0.05).

**Table 1:** Baseline characteristics of the heart failure patients

Terms	MI (n = 18)	HF(n = 24)	*P-*value
Age, years	57.83 ± 9.28	64.38 ± 10.89	*, 0.047
Male/female	15(83.33%)	17(70.83%)	
STEMI/NSTEMI	12(66.67%)	16(66.67%)	
Thrombolytic therapy	3(16.67%)	1(4.17%)	
BNP, pg/ml	328.5 ± 299.842	3535.96 ± 3025.35	****,<0.0001
CK	755.56 ± 916.38	884.71 ± 1006.39	0.6714
CK-MB	78.17 ± 87.90	98.79 ± 129.06	0.563
Glucose, mmol/L	6.62 ± 2.63	7.36 ± 3.36	0.4461
TC, mmol/L	5.59 ± 1.51	4.69 ± 1.13	*, 0.0312
TG	2.04 ± 1.45	1.47 ± 0.82	0.1153
LDL-c, mmol/L	3.51 ± 1.03	3.02 ± 0.65	0.0681
WBC	10.49 ± 3.49	11.09 ± 2.30	0.5094
AST	87.61 ± 78.98	110.83 ± 112.36	0.45888
ALT	35.56 ± 19.73	33.625 ± 16.55	0.7323
SCr	73.22 ± 17.99	84.83 ± 30.69	0.1608
BUN	5.24 ± 2.15	7.20 ± 3.49	*, 0.0428
Heart rate, beats/min	76.28 ± 10.48	79.88 ± 14.10	0.3685
Smoke	16(88.89%)	14(58.33%)	
Blood pressure, mm Hg			
Systolic	128.11 ± 16.18	133.54 ± 32.03	0.5145
Diastolic	82.22 ± 12.53	78 ± 17.11	0.3824
Comorbidity			
Diabetes mellitus (yes/no)	4(22.22%)	4(16.67%)	
Hypertension (yes/no)	10(55.56%)	12(50%)	
Hypercholesterolemia(yes/no)	11(61.11%)	12(50%)	

Data presented as mean (standard deviation), median (interquartile range), or n (%)

**Table 2:** Potential biomarkers in serum of heart failure patients

No.	Metabolite	VIP	*P*-value	Fold Change	Type
1	CL(78:6)	1.117	2.65E-04	1.443	up
2	PC(22:4_14:1)	3.007	1.54E-04	1.292	up
3	WE(17:0)	2.978	2.32E-03	1.255	up
4	WE(19:0)	1.772	1.01E-02	1.217	up
5	SPH(d19:0)	1.759	1.08E-02	1.211	up
6	phSM(t38:6)	1.656	3.26E-02	1.174	up
7	SM(d38:4)	1.126	6.42E-03	1.169	up
8	PC(36:5)	1.271	3.11E-02	1.162	up
9	TG(18:3e_17:1_20:5)	1.481	5.31E-03	1.158	up
10	TG(18:1_18:2_18:3)	2.949	1.28E-02	1.150	up
11	TG(12:1e_22:6_22:6)	1.072	1.96E-02	1.130	up
12	TG(15:0_18:2_20:5)	1.975	3.39E-02	1.097	up
13	PC(16:1_18:1)	1.619	4.81E-02	0.883	down
14	PC(16:0_20:3)	2.314	1.22E-02	0.844	down
15	PC(16:0_20:3)	1.225	7.03E-03	0.841	down
16	Hex1Cer(t38:2 + O)	1.576	3.89E-04	0.836	down
17	TG(16:0_18:1_18:3)	4.139	2.62E-02	0.831	down
18	PI(18:0_20:4)	1.512	4.84E-03	0.825	down
19	TG(18:1_18:1_20:4)	2.157	4.42E-02	0.820	down
20	PC(36:2)	8.414	8.54E-05	0.812	down
21	TG(18:1_18:1_18:2)	3.369	1.04E-02	0.806	down
22	TG(18:1_18:1_18:1)	3.868	1.27E-02	0.803	down
23	PE(14:1e_23:1)	1.622	2.78E-02	0.801	down
24	PC(18:1_18:1)	2.212	6.62E-04	0.798	down
25	PC(18:0_16:0)	6.634	7.31E-05	0.796	down
26	LPC(18:0)	1.110	1.00E-02	0.789	down
27	PE(14:1e_23:0)	2.294	2.50E-02	0.789	down
28	SM(d20:0_24:4)	1.109	6.98E-03	0.786	down
29	phSM(t38:2)	1.615	1.10E-04	0.779	down
30	PC(18:0_18:1)	2.226	5.86E-03	0.769	down
31	Hex1Cer(t41:6)	2.366	1.04E-03	0.768	down
32	LPC(18:2)	1.013	4.93E-02	0.766	down
33	PC(18:0_20:3)	3.418	5.25E-03	0.761	down
34	TG(18:1_17:1_18:2	1.333	8.10E-03	0.756	down
35	TG(16:0_18:2_22:5)	2.626	1.13E-02	0.749	down
36	TG(20:1_18:1_18:2)	1.705	6.79E-03	0.747	down
37	PC(36:1e)	2.128	1.90E-02	0.746	down
38	LPC(20:3)	1.431	7.69E-03	0.735	down
39	TG(16:0_16:1_18:2)	4.056	3.24E-02	0.734	down
40	SM(d42:1)	2.105	2.99E-03	0.734	down
41	PC(8:1e_10:1)	2.630	4.17E-02	0.733	down
42	TG(18:1_18:1_18:3)	5.550	4.14E-03	0.732	down
43	LPC(20:5)	1.084	1.57E-02	0.729	down
44	PC(8:1e_12:4)	1.133	1.42E-02	0.721	down
45	Cer(m18:1_24:0 + O)	1.146	4.63E-03	0.718	down
46	Cer(d18:1_24:0)	1.114	5.95E-03	0.712	down
47	PC(34:3)	1.447	2.41E-02	0.712	down
48	phSM(t40:3)	2.239	1.14E-04	0.708	down
49	TG(18:2_17:1_18:2)	1.038	5.90E-03	0.703	down
50	PE(39:2e)	1.699	1.85E-03	0.703	down
51	PC(20:4e_24:0)	1.174	2.42E-03	0.698	down
52	DG(18:1_18:2)	1.421	1.86E-02	0.696	down
53	PC(20:2_18:2)	2.960	3.97E-03	0.696	down
54	Co(Q10)	1.496	2.06E-03	0.692	down
55	PC(36:0)	8.042	1.90E-03	0.683	down
56	phSM(t40:3)	1.427	1.55E-03	0.662	down
57	TG(16:0_16:0_19:0)	3.522	2.77E-03	0.657	down
58	TG(18:3_18:2_20:4)	1.005	4.29E-02	0.646	down
59	TG(16:1_18:1_18:3)	6.524	3.70E-03	0.635	down
60	PE(18:2e_20:4)	1.097	7.22E-04	0.633	down
61	PE(18:1p_18:1)	1.210	5.73E-04	0.626	down
62	PE(18:1e_20:4)	1.529	3.06E-03	0.623	down
63	PE(16:0p_20:4)	1.149	9.76E-03	0.621	down
64	TG(18:1_14:0_18:3)	3.837	8.90E-03	0.619	down
65	TG(18:1_18:2_18:3)	5.848	8.69E-03	0.618	down
66	PE(16:1e_20:4)	1.063	2.22E-03	0.614	down
67	PE(18:1p_20:4)	1.553	7.77E-04	0.607	down
68	phSM(t38:3)	1.486	6.28E-03	0.590	down
69	TG(16:1_18:2_18:3)	3.005	5.31E-03	0.584	down
70	PE(18:1p_18:2)	1.173	3.35E-06	0.569	down
71	PC(16:0_18:1)	1.129	1.87E-02	0.567	down
72	PE(18:0p_20:4)	2.031	2.09E-03	0.567	down
73	TG(18:1_12:0_18:2)	2.089	3.64E-02	0.547	down
74	TG(18:3_18:2_18:3)	1.311	1.82E-02	0.544	down
75	TG(12:0_18:2_18:2)	1.182	2.14E-02	0.536	down
76	TG(18:2_14:1_18:2)	2.116	1.02E-02	0.528	down
77	PE(12:1e_22:0)	1.366	9.80E-07	0.498	down
78	TG(10:0_18:1_18:1)	1.231	4.57E-02	0.495	down
79	TG(6:0_11:1_18:2)	1.192	4.47E-03	0.471	down
80	PC(38:7)	1.062	6.84E-03	0.446	down
81	PC(38:5e)	1.979	8.44E-04	0.437	down
82	TG(18:3_18:2_20:5)	1.931	9.17E-03	0.435	down
83	SPH(d16:0)	1.804	9.68E-04	0.403	down
84	SiE(16:0)	1.779	3.52E-06	0.396	down
85	PC(34:3)	3.710	1.11E-02	0.299	down
86	DG(32:0e)	1.489	2.07E-02	0.240	down
87	SPH(t16:0)	1.812	1.53E-03	0.142	down
88	PA(44:5)	2.468	2.49E-02	0.117	down

Abbreviations: Phosphatidylcholine (PC), Phosphatidylethanolamine (PE), Lysophosphatidylcholine (LPC), Phosphatidate (PA), Sphingomyelin (SM), Ceramide (Cer), Cyclosin (CL), diglyceride (DG), Sphingosine (SPH), Triglyceride (TG), Co(Coenzyme Q10), Hexaoxacyclooctadecane (Hex1Cer), phosphatidylinositol (PI).

### Metabolomics analysis

#### Lipid composition analysis

**Fig. 1:**
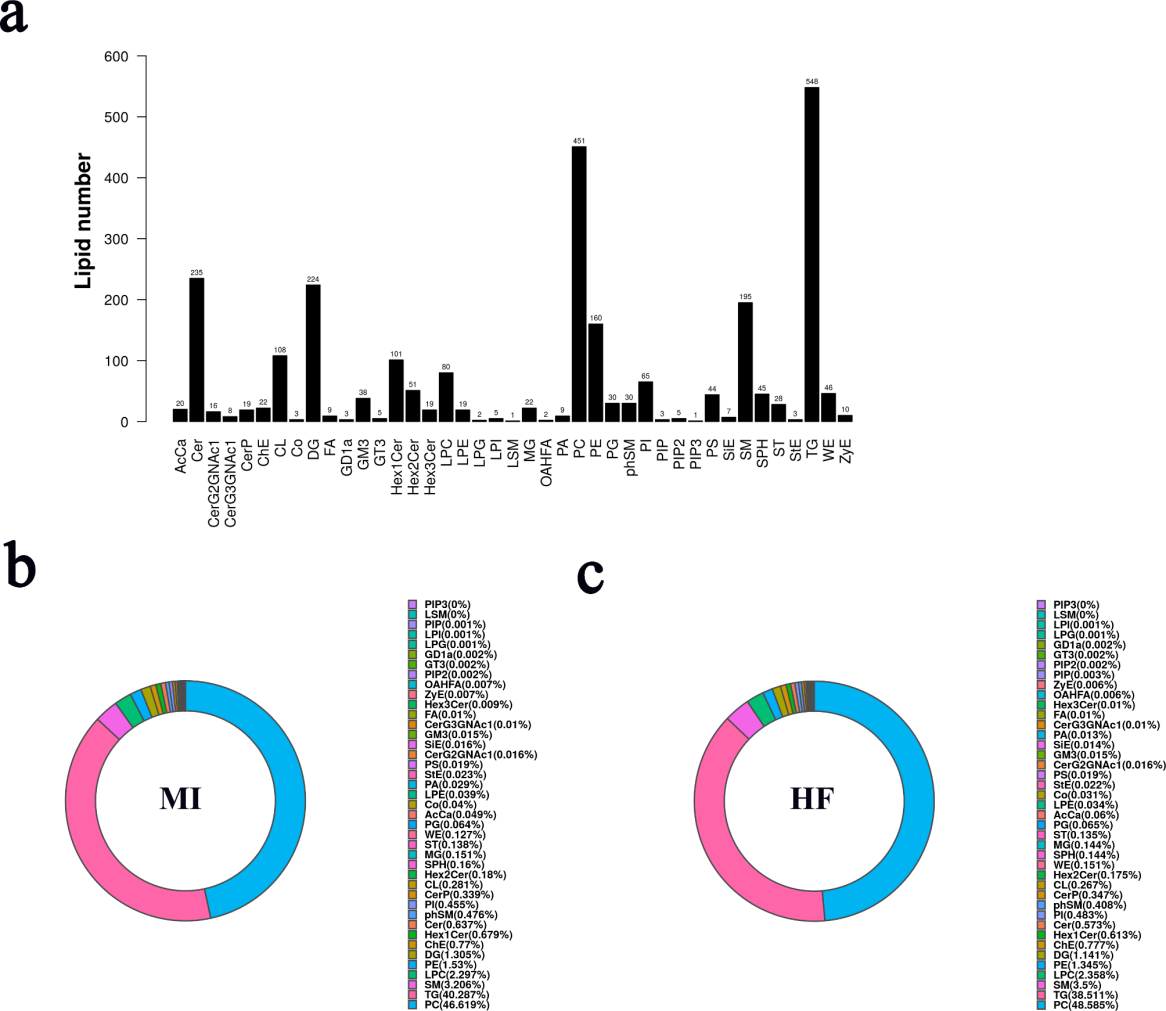
Lipid subclass **(a)** molecular numbers and lipid subclass composition of patients with **(b)** post-myocardial infarction heart failure (pMIHF) and **(c)** myocardial infarction (MI).

**Fig. 2:**
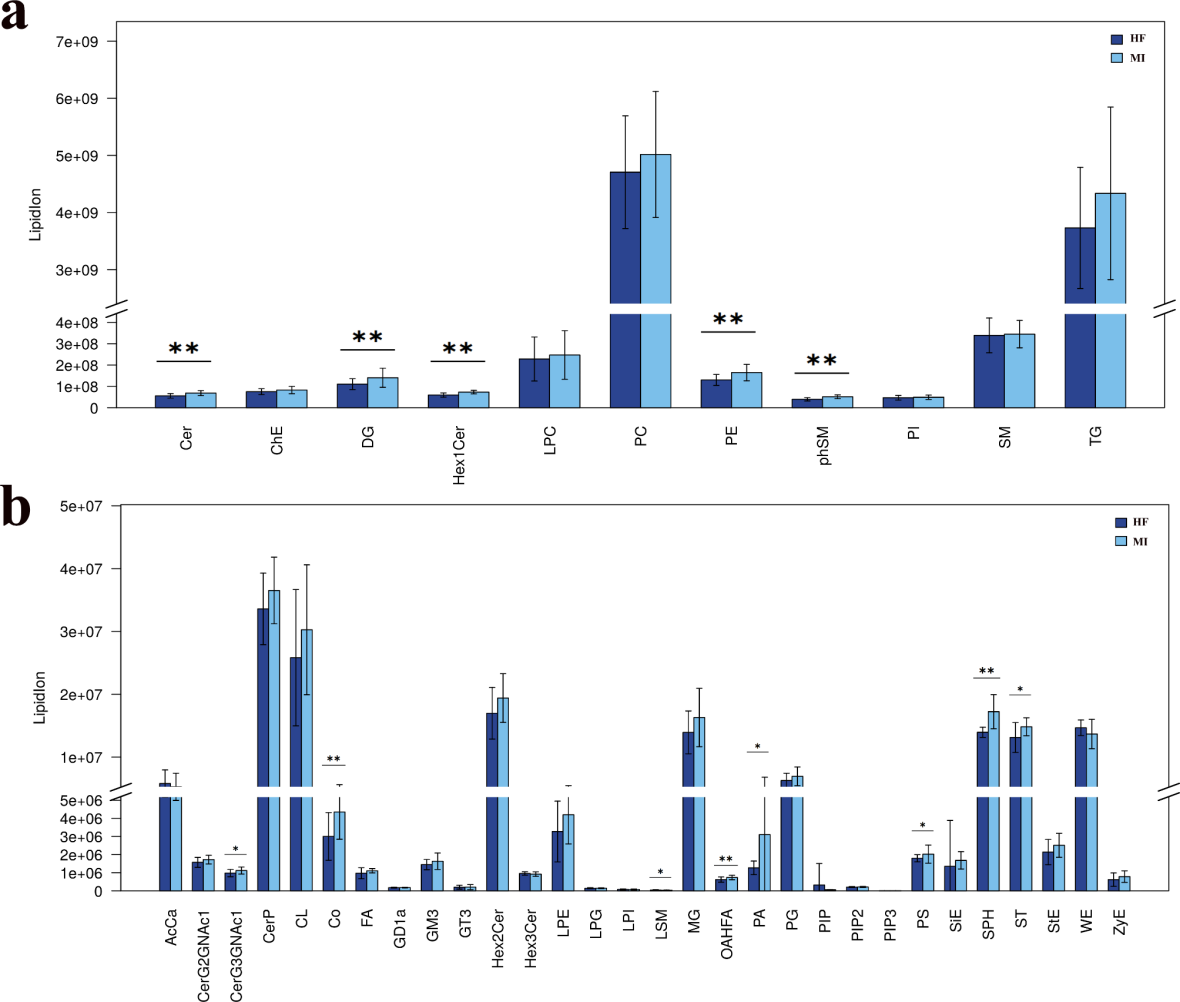
**Differences of lipid subclass levels in serum between patients with post-myocardial infarction heart failure (pMIHF) and myocardial infarction (MI).** (Compared to MI, P^*^<0.05, P^**^<0.01, MI = 18, pMIHF = 24)

The study detected 2,692 lipid species in all participants (Fig. 1a). The lipid subclass composition in patients with MI and pMIHF was dominated by PC and triglycerides (TG) (Fig. 1b). The detailed lipid subclass content (Fig. 2) showed that 12 lipid subclasses, including PE, DG, Cer, phSM, Hex1Cer, G3GNAc1, Co, OAHFA, PA, PS, SPH, and ST were significantly decreased (*p* < 0.01 or < 0.05) compared to those in the MI patients, whereas LSM was significantly increased (*P* < 0.05). These results indicate that these decreased and increased lipids can be potential biomarkers for pMIHF. Therefore these lipid subclasses should be focused on and further verified.

#### Multivariate statistical analysis

**Fig. 3:**
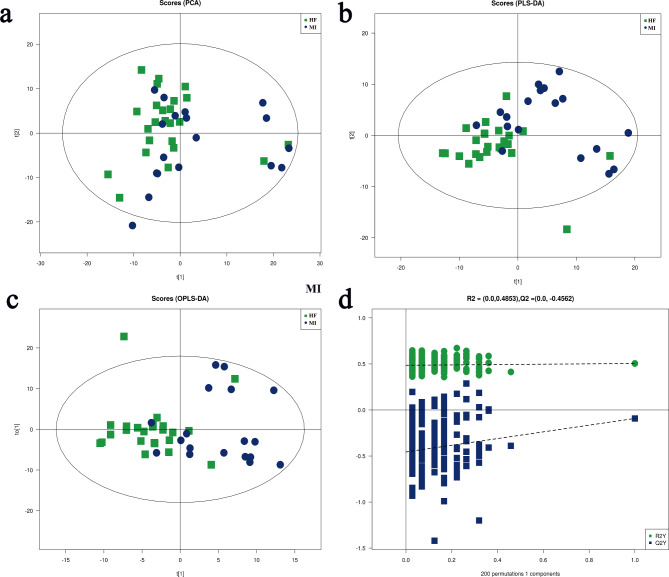
Multivariate statistical analysis between post-myocardial infarction heart failure (pMIHF) and myocardial infarction (MI). **(a)** Principal component analysis (PCA) Plot, **(b)** partial least-squares discriminant analysis (PLS-DA) Plot, **(c)** orthogonal projections to latent structures-discriminate analysis (OPLS-DA) score Plot, and **(d)** permutation test (200 cycles) of the MI group and HF group (MI = 18, pMIHF = 24).

Metabolic profile changes were analyzed to observe the overall metabolic impact on the serum of patients with MI and pMIHF. First, the PCA score plot (Fig. 3a) showed that the metabolic profile of the serum of patients with pMIHF was disturbed when compared to that of patients with MI. Additionally, the internal group was likely to be aggregated, exhibiting a robust prediction performance; and there was no overfitting, suggesting that HF can induce metabolic disorders. Next, the OPLS-DA score plot (Fig. 3b) revealed a separation of the pMIHF group from the MI group. The samples from pMIHF patients appeared to be more gathered, suggesting that there were serious lipid metabolic disorders patients with pMIHF.

#### Screening of potential biomarkers

The OPLS−DA score plot (Fig. 3c) showed that the serum samples of the pMIHF group were more clustered and could be clearly distinguished from the MI group. In the permutation tests (Fig. 3d), on the left, the R2 and Q2 values were lower than those on the right. The R2 was 0.0483 while the Q2 was − 0.4652, indicating that the models were available for further identification. The study further validated 88 potential biomarkers using the Shanghai Applied Protein Technology Inc. databases with VIP > 1 and *P* < 0.05 (Table 2). Among these, since the overall potential biomarkers were downregulated in pMIHF patients, other further validated potential biomarkers indicated that 19 PCs (90.48%), 23 TGs (85.19%), and 12 PEs (100%) were downregulated in patients with pMIHF, suggesting that PC, TG, and PE may be novel biomarkers in pMIHF diagnosis.

**Fig. 4:**
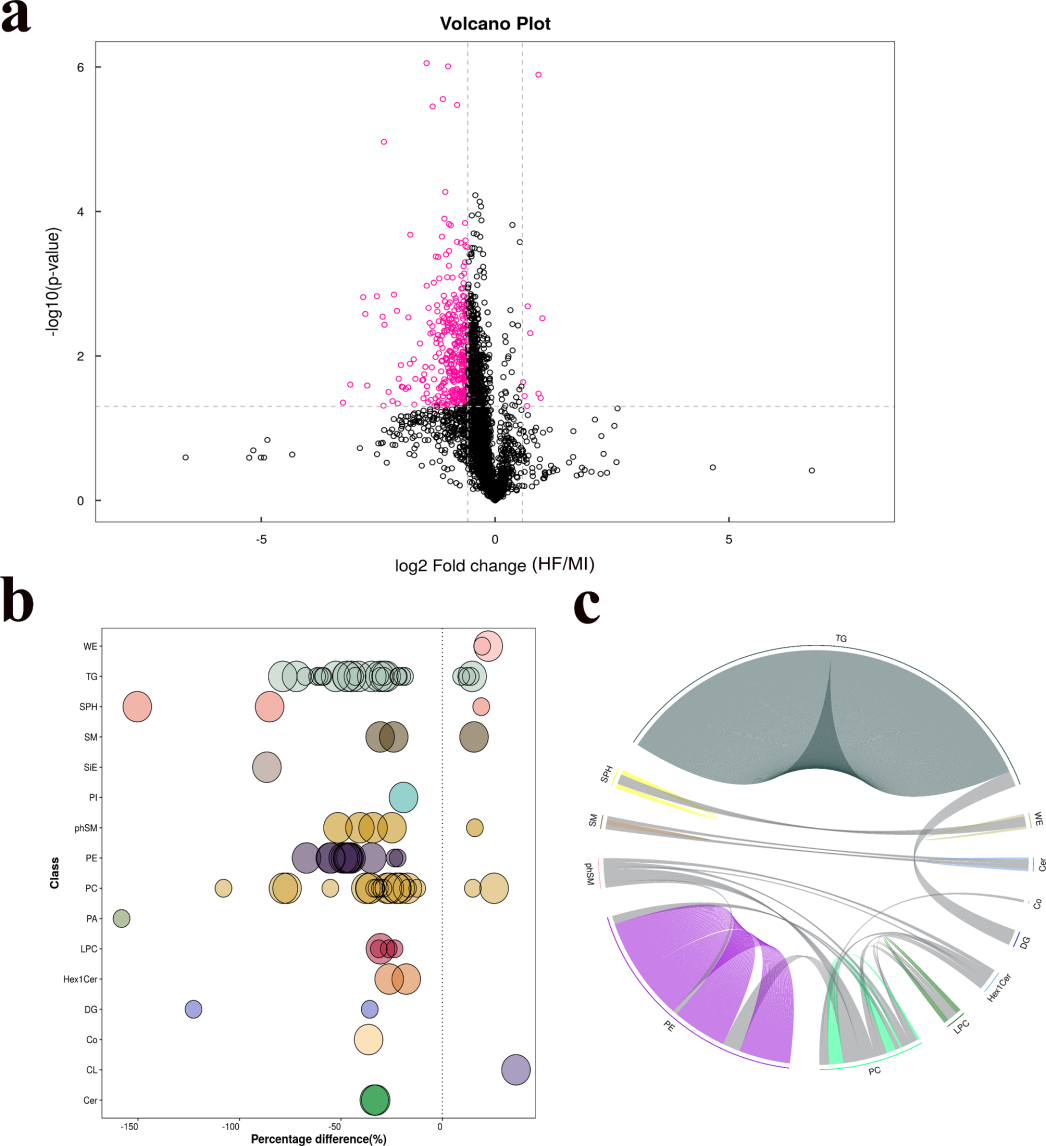
Potential biomarkers visualization analysis in post-myocardial infarction heart failure (pMIHF). **(a)** volcano plot (potential biomarkers fold change(FC)>1.5 or FC<0.67, and *P*<0.05 were presented in red), **(b)** differences of lipid subclass bubble plot (bubble size represents the significance of the difference), and **(c)** chord plot of lipid subclass

**Fig. 5:**
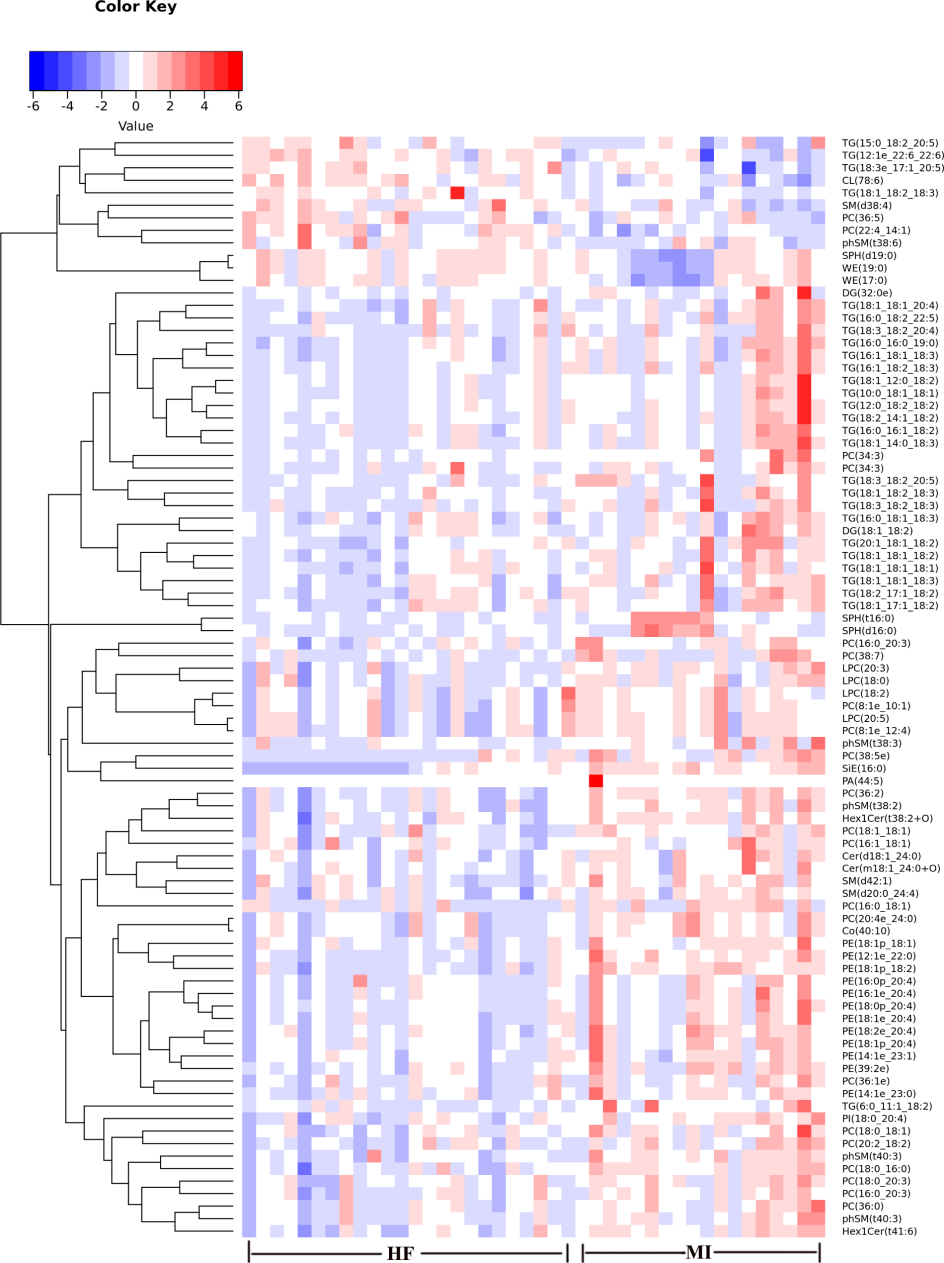
**Dysregulated lipids revealed by lipidomics in post-myocardial infarction heart failure (pMIHF).** (Rows: potential biomarker; columns: samples; red color represents high metabolite content, while blue refers to low metabolite content)

Figure 4a shows a volcano plot displaying 12 upregulated and 76 downregulated potential biomarkers. Among them, the content differences for each lipid subclass are represented in the bubble plot (Fig. 4b). Larger differences are indicated by large bubbles, PE and PC appeared to be more gathered. Next, the correlation analysis revealed that PC was most closely associated with other lipid subclasses, followed by PE (Fig. 4c). To better visualize the overall trend of potential biomarkers in the samples between pMIHF and MI groups, clustering analysis was performed to illustrate the changing trends in potential biomarker contents (Fig. 5). The results obtained suggested that most potential biomarkers decreased in patients with pMIHF, especially in PE, PC, and TG.

#### Metabolic pathway analysis

**Fig. 6:**
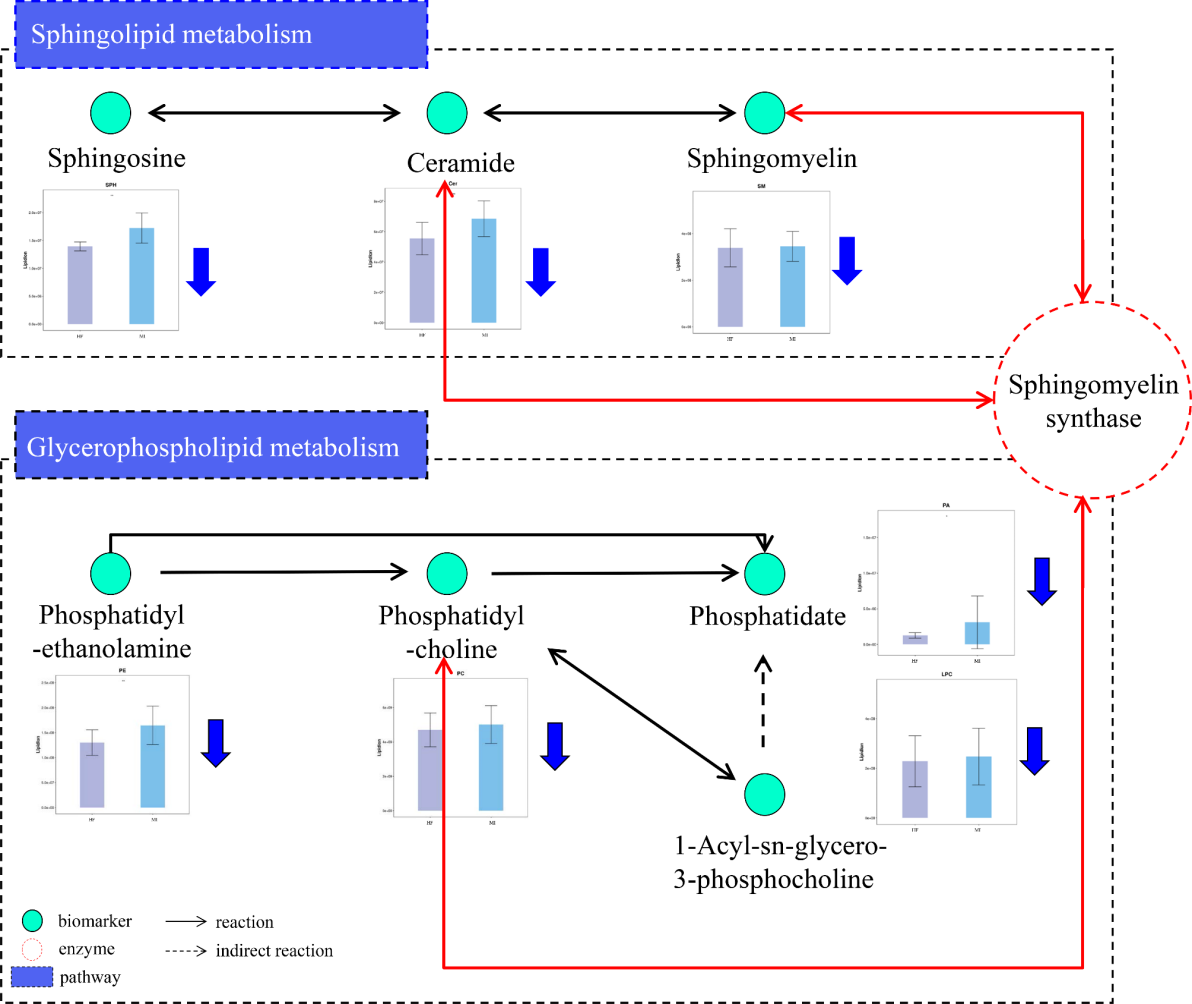
Brief lipid metabolism pathways with post-myocardial infarction heart failure (pMIHF) patients

Pathway enrichment showed that eight metabolic pathways were disturbed in patients with pMIHF, and potential biomarkers were identified, including PE, PC, and Cer (Table 2). Among these pathways, glycerophospholipid and sphingolipid metabolism (*P* < 0.05) were identified as core pathways in pMIHF with higher impact values (> 0.1). Seven decreased lipid subclasses were involved in the above metabolic pathways (Fig. 6). In sphingolipid metabolism, Sphingosine (SPH), Ceramide (Cer), and Sphingomyelin(SM) were decreased, further implying that Cer and SM could inhibit sphingomyelin synthase metabolism. In glycerophospholipid metabolism, PE, PC, phosphatidate (PA), and 1-Acly-sn-glycero-3-phosphochline (LPC) were decreased, PC could inhibit sphingomyelin synthase metabolism. The results revealed that the inhibition of lipid metabolism may play a key role in pMIHF disease progression, suggesting that downregulated lipids metabolites may be a novel biomarker in pMIHF diagnosis.

### Integrative analysis of biomarkers

**Fig. 7:**
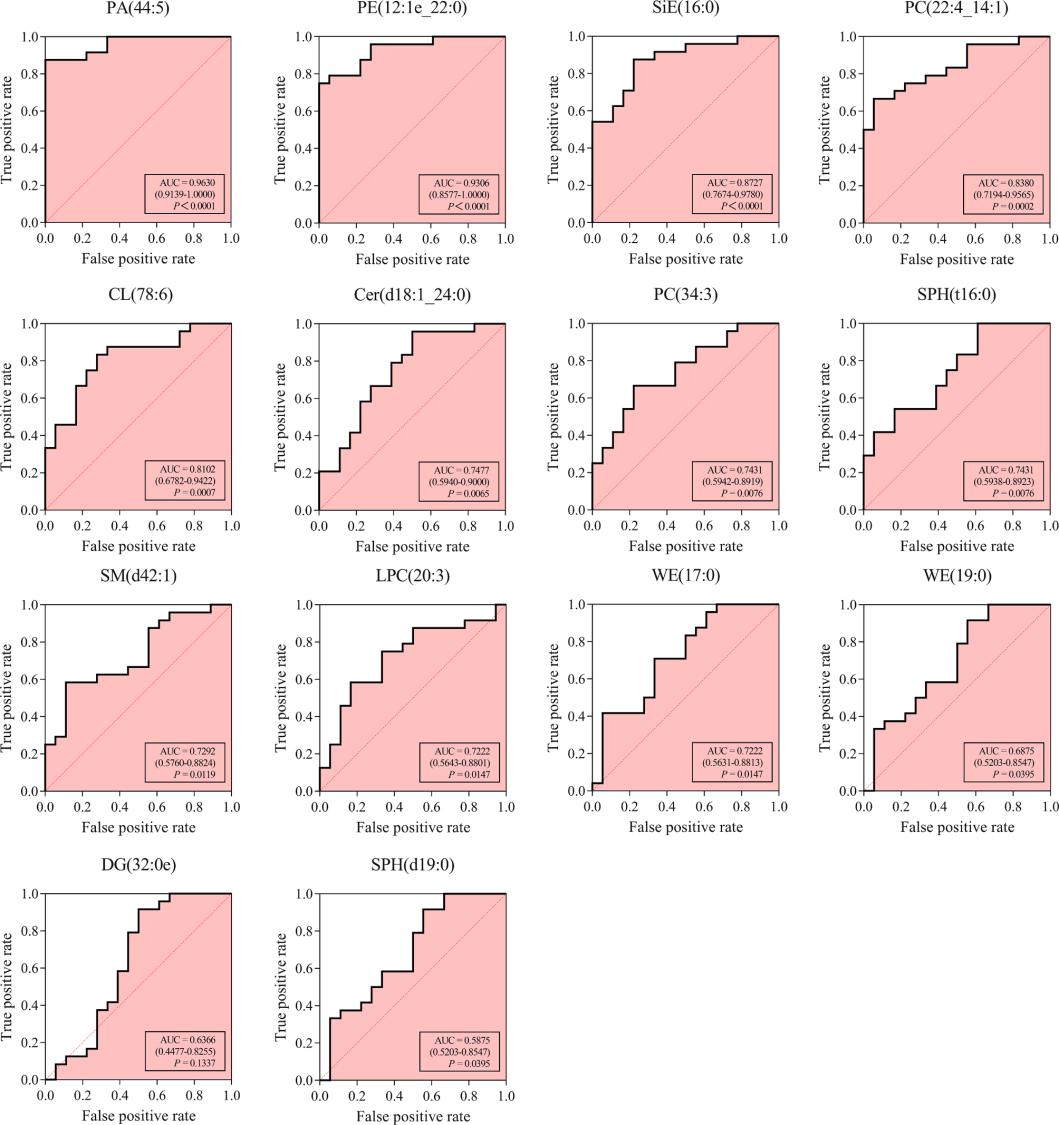
**ROC analysis revealed candidate biomarkers for post-myocardial infarction heart failure (pMIHF) diagnosis.** (AUC, 95% confidence intervals, grouped scatter plots, and cutoff values are also shown)

Due to the inhibition of lipid metabolism, a total of 14 potential biomarkers involved in glycerophospholipid and sphingolipid metabolism were analyzed for further verification. Relatively high AUC (A) values showed excellent predictive power for potential biomarkers in patients with pMIHF. ROC curves (Fig. 7) indicated that phosphatidate (PA; 44:5; A = 0.9630), PE (12:1e_22:0; A = 0.9306), SiE (16:0; A = 0.8727), PC (22:4_14:1; A = 0.8380), cyclosin (CL(78:6); A = 0.8102), Cer (d18:1_24:0; A = 0.7477), PC(34:3; A = 0.7431), SPH(t16:0; A = 0.7431), SM(d42:1; A = 0.7292), and LPC(20:3; A = 0.7222) were the top ten potential biomarkers with the highest A values.

**Fig. 8:**
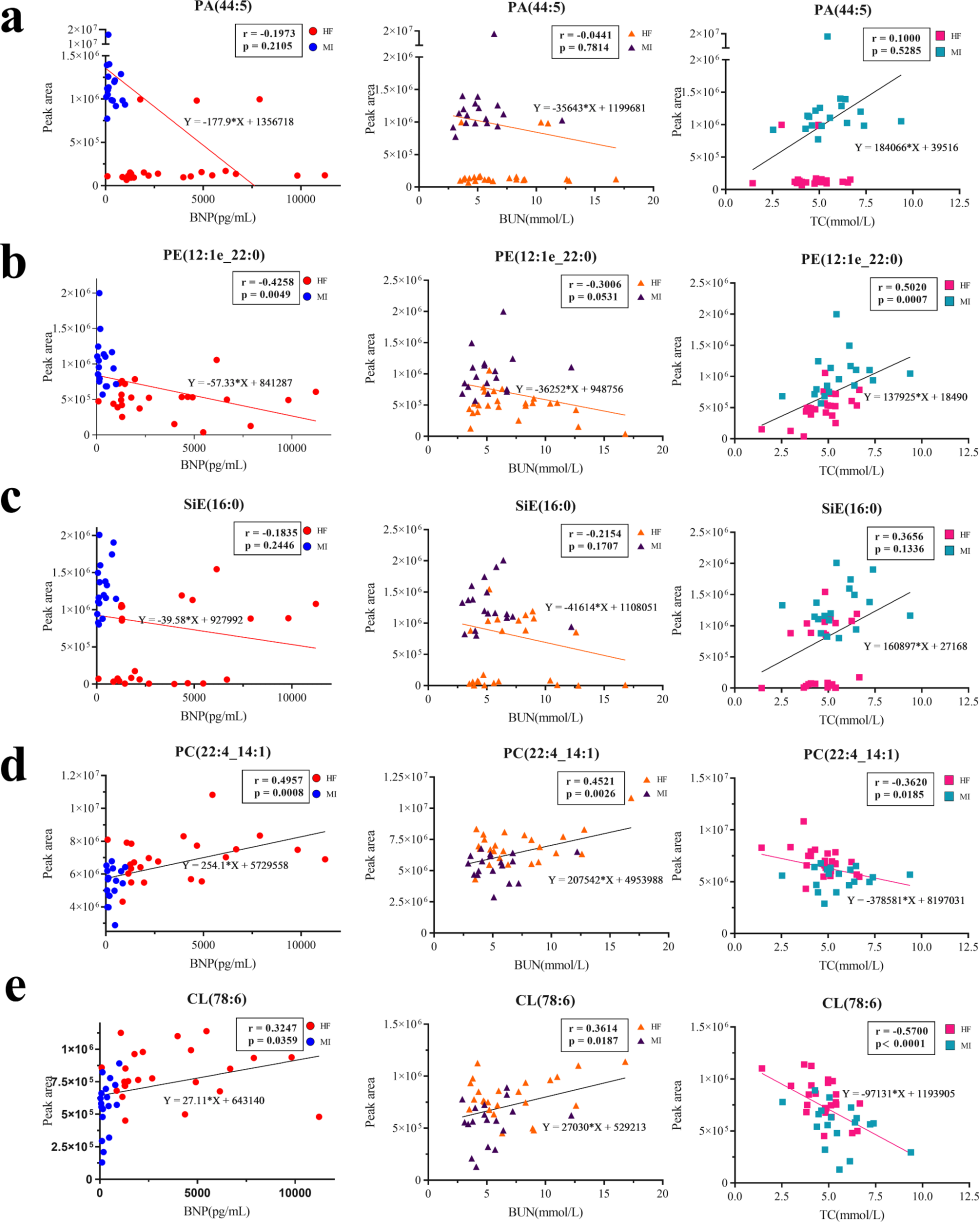
**Correlation analysis of biochemical indicators and dysregulated lipids in post-myocardial infarction heart failure (pMIHF) patients.** a–e Scatter plots presenting the relationship between the BNP/BUN/TC and the intensity of PA, PE, SiE, PC, and CL. (Pearson r and *P* value were shown. Groups were presented in different colors)

Then top five potential biomarkers were used for correlation analysis (Fig. 8). The results demonstrated that most of these components were negatively correlated. Of these, PC (22:4_14:1) and CL (78:6) were positively correlated with BNP (r = 0.4957 and r = 0.3247, respectively) and BUN (r = 0.4521 and r = 0.3614, respectively), but negatively correlated with TC (r = − 0.3620, r = − 0.5700). In contrast, PE (12:1e_22:0), PA (44:5), and Sie (16:0) were positively correlated with TC (r = 0.5020) and negatively correlated with BNP (r = − 0.4258) and BUN (r = − 0.3006). However, PA (44:5) and Sie (16:0) had no statistical significance. Notably, PE and PC are involved in glycerophospholipid metabolism, which may play a key role in pMIHF disease progression. In summary, the biomarker panels for PE (12:1e_22:0) and PC (22:4_14:1) could sufficiently differentiate between patients with MI and pMIHF when analyzed using ROC and correlation analysis.

## Discussion

The mechanisms that lead to pMIHF are currently being elucidated. Considering the unique role of lipid disorders in the pathogenesis of pMIHF, there is substantial interest in understanding the changes in lipid metabolism pathways as this may aid in biomarker screening. Accordingly, lipidomics assays were used to delineate the metabolic profiles of numerous lipids and elucidate the lipid characteristics associated with pMIHF, thus, providing reference data for the identification of early diagnostic biomarkers. pMIHF disease lacks early biomarkers for diagnosis, and lipidomics analysis of pMIHF serum samples has not confirmed any novel biomarkers. There is an urgent need to discover new lipid markers that can enable the early diagnosis of pMIHF and provide a powerful aid in the clinical management and prognosis of patients with pMIHF. In this study, 18 MI patients and 24 pMIHF patients were subject to metabolic profile analysis to determine potential biomarkers and metabolic pathway disorders. ROC and correlation analysis were utilized to analyze the metabolic profile results and indicate potential biomarkers for pMIHF diagnosis.

### Baseline characteristics analysis

In this study, 42 subjects were included in the serum lipidomic analysis. The baseline characteristics demonstrated that patient age and BNP, BUN, and TC levels were significantly different between the MI and pMIHF groups, indicating that elderly patients with MI and renal function impairment may be more susceptible to HF.

Aging is a major risk factor for HF, and a sizeable percentage of elderly patients with HF have cardiac amyloidosis, which is an HF precipitator [19, 20]. BNP is an endogenous cardiac peptide and an established HF predictor, whose levels are proportional to the severity of HF [21]. In addition, patients with HF should be monitored for changes in BNP levels over the first month after discharge since they are a useful prognostic indicator of rehospitalization [22] [23]. Relatively high BNP levels in chronic kidney disease is a major player in the heart-renal connection [24]. Our results demonstrated that BNP and BUN levels are elevated in patients with pMIHF. Conversely, TC has been suggested to increase the risk of CAD [25], and our results showed that TC levels were low in patients with pMIHF. Furthermore, low serum TC levels can be a predictor of adverse outcomes that disproportionately affect advanced HF patients [26–28]. Increased BNP and BUN levels are commonly used as indicators to predict HF. Therefore, a reduction in TC can also serve as a prognostic indicator of the dynamic changes occurring during pMIHF development.

### Lipid disorders

A total of 88 lipids, including 76 (86.36%) downregulated lipids, were detected between 18 patients with MI and 24 patients with pMIHF. Lipids are inhibited in patients with pMIHF, leading to disorders in glycerophospholipid and sphingolipid metabolism. The total levels of PE, PC, and TG were decreased in patients with pMIHF.

Lipids are a critical component of the membrane and play an essential role in energy storage and metabolism [29]. TG-rich lipoprotein are known to cause lipotoxic cardiomyopathy and cardiac steatosis [30]. Progressively higher TG concentrations are associated with a progressively higher risk of HF and MI [31, 32]. PC is a key source of bioactive eicosanoids, including leukotrienes, prostaglandins, and lipoxins, which increase free fatty acid accumulation and inflammation. Mammalian cells mainly contain Pes in their plasma and mitochondrial membranes [33]. Molecular PC/PE ratios influence mitochondrial energy metabolism and contribute to disease progression in cells [34]. In the present study, 19 PCs (90.48%), 23 TGs (85.19%), and 12 PEs (100%) were downregulated, which may contribute to lipotoxic cardiomyopathy, disruptions to mitochondrial function, and inflammation in patients with HF and subsequent MI-associated lipotoxic cardiomyopathy.

### Potential lipid biomarkers

To better screen for early warning biomarkers of pMIHF, several lipid biomarkers were identified for ROC analysis. Based on the ROC results, significant biomarkers were further subjected to correlation analyses with TC, BUN, and BNP levels. PA (44:5), PE (12:1e_22:0), SiE (16:0), PC (22:4_14:1), and CL (78:6) showed higher AUC values. PAs are bioactive lipids that are activated by a variety of inflammatory mediators, including bradykinin, ATP, and glutamate; PAs also contribute to the modulation of cardiac contractility [35]. Our results showed that PA lipid content was the lowest in patients with pMIHF.

Correlation analysis showed that PC (22:4_14:1), PE (12:1e_22:0), and CL (78:6) were strongly correlated with TC, BUN, and BNP. Notably, PC and PE are involved in key glycerophospholipid and sphingolipid metabolism pathways. PC disorders can disturb myocardial metabolism and cellular signaling [36]. Thus, PCs, particularly, the metabolism of PCs in the failing heart may be associated with alterations in myocardial lipid homeostasis [37]. PC (20:0/18:4), PC (20:4/20:0), PC (40:4), PC (20:4/18:0),and PC (34:4) were increased, whereas PC(32_0), PC(C34:4), and PC (36:5) were decreased in HF, thus giving PC a diagnostic value for HF similar to that of BNP [11, 38, 39]. PE levels are significantly increased in serum and cardiac samples during HF [16, 40], and PE (O-18:1(1Z)/20:4(5Z,8Z,11Z,14Z)) levels can be improved by higenamine in doxorubicin-induced HF rats [41]. This study observed the upregulation of PC (22:4_14:1) and downregulation of PE (12:1e_22:0), which exhibited a close correlation with the indicators of HF, including BNP. Therefore, PC and PE may be key potential biomarkers in patients with HF with subsequent MI. The study also showed a positive correlation between pMIHF and mortality among patients with MI.

It is important to keep in mind that optimal management of patients with pMIHF depends on the time since their infarction began. Patients with pMIHF show poor prognosis. An observational study of 4,825 in-hospital deaths among patients who suffered non-ST-segment elevation MIs in Canada from 1999 to 2003 found that the presence of HF on admission significantly increased in-hospital mortality [42]. HF complicated by MI requires early intervention owing to its high mortality rate. However, early detection is hindered due to the lack of accurate biomarkers for the disease. This makes the treatment challenging. There are two emerging biomarkers for ischemic cardiomyopathy: angiopoietin-2 and thrombospondin-2, that are now used in addition to BNP and cardiac troponin [43]. Additionally, miRNAs contribute significantly to the exploration of novel pMIHF biomarkers for future applications [44]. Higher circulating LIPCAR levels have been found in patients with pMIHF [45]. Our study has revealed several specific lipid disorders in patients that were highly related to clinical diagnosis indicators. The emergence of lipidomics tools have rapidly improved the process of discovery of biomarkers. Thus, the upregulation of PC (22:4_14:1) and downregulation of PE (12:1e_22:0) provides a novel perspective from which to explore potential serum lipid biomarkers in patients.

### Strengths and limitations

This study has several strengths. First, the study successfully obtained potential serum lipid profiles using the UHPLC-Q-Exactive MS lipidomics approach, to screen the metabolic differences between patients with MI and pMIHF, and combine this analysis with ROC analysis. Second, correlation analysis revealed that PC (22:4_14:1) and PE (12:1e_22:0) were highly correlated with BNP, and could, thus, be used as novel biomarkers for early heart failure diagnosis.

This study also had several limitations. First, targeted lipidomic analysis was not performed. Second, the sample size of clinical samples was not sufficiently large and wasn’t externally validated. Finally, middle-aged to older people were obtained individually for detection in this study. Future studies should use integrative untargeted and targeted metabolomics and lipidomics to study potential differences between disease groups. Multicenter studies involving other races, ethnicities, and cultures within Asia are also needed with a larger sample size to corroborate our results. These findings should also be externally validated to identify their generalizability by combining proteomics, metabolomics, lipidomics, and urine analysis to specifically identify potential biomarkers.

## Conclusions

This study revealed distinct lipid metabolites and lipidomic patterns in an independent cohort that were significantly associated with future risk of developing pMIHF. The results of this study suggest that PC (22:4_14:1) and PE (12:1e_22:0) are potential predictors for the development of HF after MI. Thus, the study provided a new strategy for the prevention of HF by identifying biomarkers for the development of HF after MI. The search for and identification of metabolic pathways and biomarkers reflecting early changes after MI will not only help to enhance the understanding of the developmental mechanisms of HF after MI, but also provide a theoretical basis for the early diagnosis, risk prediction, and prevention strategies for cardiogenic shock.

## Data Availability

The datasets used and/or analyzed during the current study are available from the corresponding author on reasonable request.

## Abbreviations

AUC: Area under the curve
BNP: B-type natriuretic peptide
CAD: Coronary artery disease
Cer: Ceramide
CL: Cyclosin
ESI: Electrospray ionization
HF: Heart failure
LPC: Lysophosphatidylcholine
LVEF: Left ventricular volume ejected per beat
MI: Myocardial infarction
OPLS−DA: Orthogonal projections to latent structures-discriminate analysis
PA: Phosphatidate
PC: Phosphatidylcholine
PCA: Principal component analysis
PCI: Percutaneous coronary intervention
PE: Phosphatidylethanolamine
PLS-DA: Partial least-squares discriminant analysis
pMIHF: Post-MI-heart failure
ROC: Receiver operating characteristic
UHPLC-Q-Exactive-MS: Ultra-high performance liquid chromatography plus Q-Exactive mass spectrometry
VIP: Variable importance in the projection
